# Three-dimensional bioprinting using self-assembling scalable scaffold-free “tissue strands” as a new bioink

**DOI:** 10.1038/srep28714

**Published:** 2016-06-27

**Authors:** Yin Yu, Kazim K. Moncal, Jianqiang Li, Weijie Peng, Iris Rivero, James A. Martin, Ibrahim T. Ozbolat

**Affiliations:** 1 Harvard Medical School, Harvard University, Cambridge, MA, USA; 2 The Center for Engineering in Medicine, Massachusetts General Hospital, Boston, MA, USA; 3 Engineering Science and Mechanics Department, The Pennsylvania State University, State College, PA, USA; 4 Huck Institute of Life Sciences, The Pennsylvania State University, State College, PA, USA; 5 Industrial and Manufacturing Systems Engineering, Iowa State University, Ames, IA, USA; 6 Department of Orthopaedics and Rehabilitation, The University of Iowa, Iowa City, IA, USA

## Abstract

Recent advances in bioprinting have granted tissue engineers the ability to assemble biomaterials, cells, and signaling molecules into anatomically relevant functional tissues or organ parts. Scaffold-free fabrication has recently attracted a great deal of interest due to the ability to recapitulate tissue biology by using self-assembly, which mimics the embryonic development process. Despite several attempts, bioprinting of scale-up tissues at clinically-relevant dimensions with closely recapitulated tissue biology and functionality is still a major roadblock. Here, we fabricate and engineer scaffold-free scalable tissue strands as a novel bioink material for robotic-assisted bioprinting technologies. Compare to 400 μm-thick tissue spheroids bioprinted in a liquid delivery medium into confining molds, near 8 cm-long tissue strands with rapid fusion and self-assemble capabilities are bioprinted in solid form for the first time without any need for a scaffold or a mold support or a liquid delivery medium, and facilitated native-like scale-up tissues. The prominent approach has been verified using cartilage strands as building units to bioprint articular cartilage tissue.

Given its unmatched capability and rapid advancement, three-dimensional (3D) bioprinting has drawn enormous attention and become increasingly popular for engineering functional tissues and organs^1^. As the development of 3D bioprinting techniques has progressed, great attention has been paid to adopting this cutting-edge technology for a broad spectrum of applications in medicine^2^,^3^,^4^. Three-dimensional bioprinting uses an additive manufacturing strategy, which can offer precise temporal and special control of biomaterials, cells, growth factors, and other functional components^5^,^6^,^7^. There are three major elements of bioprinting: biomimicry, which strives to reproduce the identical components of a tissue or organ^8^; biological self-assembly, which mimics embryonic organ development by using cellular organization and tissue fusion^9^; and use of mini-tissues as building blocks for the fabrication of larger constructs in anatomically-correct shapes^10^. Among these approaches, self-assembly of cellular components implements principles of embryonic development while taking advantage of 3D bioprinting techniques. It is fully biological, scaffold-free, and considered to be a promising direction for bioprinting. In contrast to scaffold-based bioprinting, where tissue development depends on cell proliferation within or on top of hydrogel or other scaffolding materials, scaffold-free bioprinting can offer relatively high cell density initially without the inclusion of biomaterials. This can facilitate the deposition of extracellular matrix (ECM) in a defined manner with better cell-to-cell interactions^11^, generation of tissues with close biomimicry^12^, preservation of cell phenotype and functionality for longer term^13^, acceleration of tissue maturation^14^, and elimination of complications with biodegradation of scaffolds^15^. Using tissue spheroids, scaffold-free bioprinting has been successfully demonstrated for the fabrication of thin tubular tissues such as blood vessels^11^ and nerve grafts^16^; however, bioprinting tissues with a scalable bioink material enabling scaffold- and mold-free bioprinting of scale-up tissues is still elusive^17^.

Here we introduce a novel scalable bioink, “tissue strands,” for scaffold-free bioprinting to facilitate rapid fabrication of biomimetically developed tissues. By using the biofabrication method presented in our recent framework study^18^, we generate tissue strands that are bioprintable, facilitate rapid fusion and maturation through self-assembly, enable bioprinting in solid form, do not need a liquid delivery medium during extrusion, and do not require a support molding structure during bioprinting for cell aggregation and fusion. These unique capabilities enable scale-up tissue constructs, which may one day enable integration of a vascular network in multiple scales for bottom-up fabrication of clinically relevant-sized organs in the near future. The schematic presented in Fig. 1 elucidates the concept of tissue bioprinting using tissue strands as a new bioink. Harvested cells are microinjected into very long tubular semi-permeable capsules, which were directly printed using a coaxial microextrusion system. Tubular capsules serve as a reservoir for aggregation of cells, leaving highly long strands of perfectly cylindrical mini-tissues after dissolving the capsules. Tissue strands are then engineered and successfully loaded into a unique print head, enabling bioprinting and rapid fabrication of tissues that can be used in regenerative medicine, drug screening, and disease modeling.

In this paper, viability test revealed minimal cell damage upon fabrication, where chondrocytes were able to maintain their metabolic activity over time, as shown by active cell proliferation and sulfated glycosaminoglycans (sGAG) production. Cartilage strands were able to undergo self-assembly by fusing to each other upon bioprinting-guided positioning. Fusion of cartilage strands started as soon as 12 hours post-printing, and was nearly completed by Day 7, demonstrating their potential for scale-up tissue fabrication. The immunohistochemistry examination showed significant expression of cartilage-specific markers at both the transcription and the protein level. Cartilage extra-cellular matrix (ECM) was heavily deposited throughout mature tissue strands over time, which demonstrated the maturation of the fabricated structure. Finally, cartilage strands were engineered, bioprinted and then implanted into *ex-vivo* osteochondral tissue defects and were able to further undergo remodeling within the host tissue.

## Results

### Fabrication of Scalable Cartilage Tissue Strands

**Figure 2:**
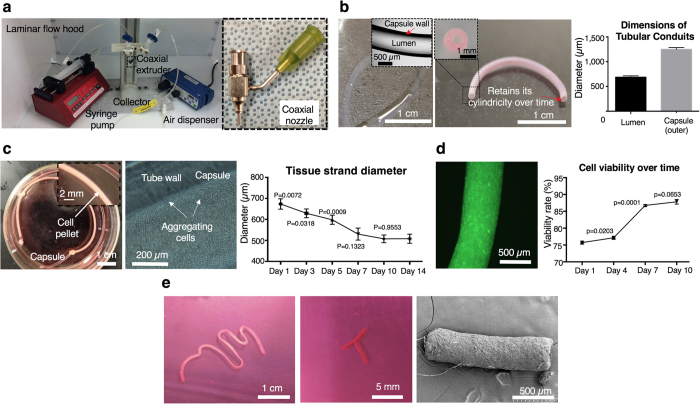
Fabrication of tissue strands. (**a)** Left: Setup for co-axial extrusion of alginate capsules. Right: A home-made coaxial nozzle apparatus. (**b)** Left: Alginate capsules with well-defined tubular morphology. Middle: Capsules preserved their cylindrical morphology without any collapse in culture over time. Right: Controlled luminal and outer diameters (*n* = 6). (**c)** Left: Microinjected cell pellet inside alginate capsules supporting aggregation in a few days. Middle: Aggregation begins around the luminal surface of the capsule. Right: Tissue strand diameter decreases as aggregation progresses due to radial contraction and stabilizes after Day 10 (*n* = 6). (**d)** Left: Viable cells (green fluorescent) under confocal microscopic view. Right: Cell viability gradually increases over a 10-day culture period (*n* = 6). (**e)** Left: A nearly 8 cm long tissue strand after decrosslinking the capsule. Middle: Tissue strands were self-assembled into a “T” shape. Right: Well-defined cylindrical morphology was achieved as shown with SEM. All data are presented as average ± SD unless otherwise stated.

Our early work demonstrated the capability of directly printing tubular microcapsules using a coaxial nozzle apparatus^19^ (Supplementary Method S1 and Supplementary Fig. 1a). In this study, we took advantage of the coaxial extrusion system (Fig. 2a, left) to assist the fabrication of tissue strands; tubular permeable alginate capsules were used as a reservoir for cell aggregation and tissue strand maturation. Using the coaxial nozzle system (Fig. 2a, right), tubular alginate capsules were successfully extruded in continuous form with highly uniform structure (Fig. 2b, left). Tubes preserved their integrity and shape in culture over time (Fig. 2b, middle). The average luminal and outer diameters of the fabricated capsules were 709 ± 15.9 μm and 1,248.5 ± 37.2 μm, respectively (Fig. 2b, right). By controlling fabrication parameters, different sizes of conduits could be fabricated (Supplementary Fig. 1b) and this would allow us to produce various sizes of tissue strands upon different request, where we have demonstrated the possibility of fabricating a few meter-long conduits with sufficient continuity and flexibility. In our earlier work^19^, we could be able to obtain tubular capsules with an outer and luminal diameter value of 639.6 ± 17.9 μm 284.2 ± 7.5 μm, respectively. Using a gas-tight microsyringe (Supplementary Fig. 2a), cell pellet (with approximately 200 million chondrocytes) was successfully microinjected into a 130 mm long microtubular capsule (Fig. 2c, left), with minimal loss of cellular material. Cells were in close contact with the inner wall of alginate capsules, which is inert to cell adhesion, thus compelling cells to develop neotissue to spontaneously adhere to one another to minimize free energy (Fig. 2c, middle). Over time, as tissue strands formed, their size started to diminish in the radial direction due to contraction, during which visible gaps were observed between the border of tissue strands and inner wall of conduits (Supplementary Methods S2 and Supplementary Fig. 2b–e). After microinjection, the average diameter of tissue strands dropped down to 639 ± 47 μm on Day 3, gradually reduced to 507 ± 18 μm on Day 10, and then did not have significant further changes, ending at 508 ± 21 μm on Day 14 (Fig. 2c, right). Tissue contraction in the radial direction can be attributed to intracellular cytoskeletal reorganization from cadherin-mediated cell-to-cell binding^20^, which occurs without the influence of external forces. In our experiments, no considerable contraction was observed longitudinally. Although cell viability was impaired at the beginning (75 ± 0.5%) due to shear stress during microinjection, cells were able to recover over time with a maintained high viability of 87 ± 3% on Day 7 (Fig. 2d). Macroscopically, tissue strands were formed with good integrity, mechanical strength and well-defined cylindrical morphology both before and after releasing them from capsules in four days. Tissue strands can be made in considerable length as long as there is sufficient cell number and we demonstrated a near 8 cm-long one in our experiments (Fig. 2e, left). Tissue strands had the ability to quickly assemble into different shapes as presented in the middle image in Fig. 2e. Stabilization in their diameter and preservation of their perfectly cylindrical shape after 10 days (Fig. 2e, right) enabled standardization of the 3D bioprinting system and ensured repeatability of the bioprinting process, respectively.

### Characterization of Cartilage Tissue Strands

**Figure 3:**
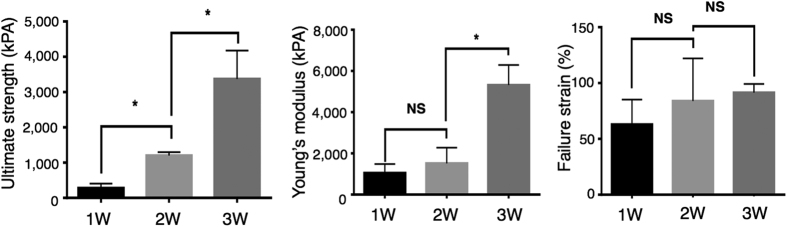
Mechanical characterization of tissue strands. Mechanical analysis of tissue strands over a three-week incubation period, including ultimate strength (left), Young’s modulus (middle) and failure strain (right) (*n* = 3 for one-week and two-week data, *n* = 4 for three-week data). All data presented as average ± SD unless otherwise stated.

To further elicit tissue strands, morphological, biological, and mechanical properties were characterized using scanning electron microscopy (SEM) (Supplementary Methods S3), biochemical assays, and mechanical testing. Scanning electron microscopy images were used to examine the ultra-structure and morphology of tissue strands periodically over a three-week culture time (Supplementary Fig. 3). Microinjected chondrocytes in high density formed compact tissue strands with tight cell-to-cell adhesion and abundant ECM deposition. Cell density gradually increased, and cells deposited fibrous ECM components longitudinally, which made them more compact and structurally integrated. Longitudinal orientation of ECM components (i.e., collagen type II fibers) can be particularly helpful in engineering heterogeneous tissues such as their orientation can be controlled during bioprinting to fabricate zonally-stratified articular cartilage with less cells and vertically-oriented collagen fibers in deeper zones, and more cells and horizontally-oriented collagen fibers in the superficial zone. Increasing structural integrity can also be confirmed by the mechanical testing results. We carried out a uni-axial tensile test to assess the mechanical properties of tissue strands at different time points. This was important during bioprinting because the tissue strands experienced tensile stress when they adhered on the bioprinting stage. As shown in Fig. 3, tissue strands showed significantly increased ultimate strength over a three-week chondrogenic culture, where the ultimate strength of one-week-cultured strands increased from 283.1 ± 70.36 kPa at one week to 1,202 ± 56.28 kPa at two weeks to 3,371 ± 465.0 kPa at three weeks. The Young’s modulus of tissue strands increased from 1,050 ± 248.6 kPa at one week to 1,517 ± 438.1 kPa at two weeks to 5,316 ± 487.8 kPa at three weeks. Although failure strain increased over time, results did not show significant differences between Week 1 (62.93 ± 12.83%), Week 2 (83.93 ± 22.03%), and Week 3 (91.46 ± 3.852%). This increase in the mechanical properties of tissue strands offers an opportunity to optimize cultivation time in order to have tissue strands engineered so that they can sustain tensile forces during bioprinting while remaining reasonably malleable for bioprinting into desired geometry.

**Figure 4:**
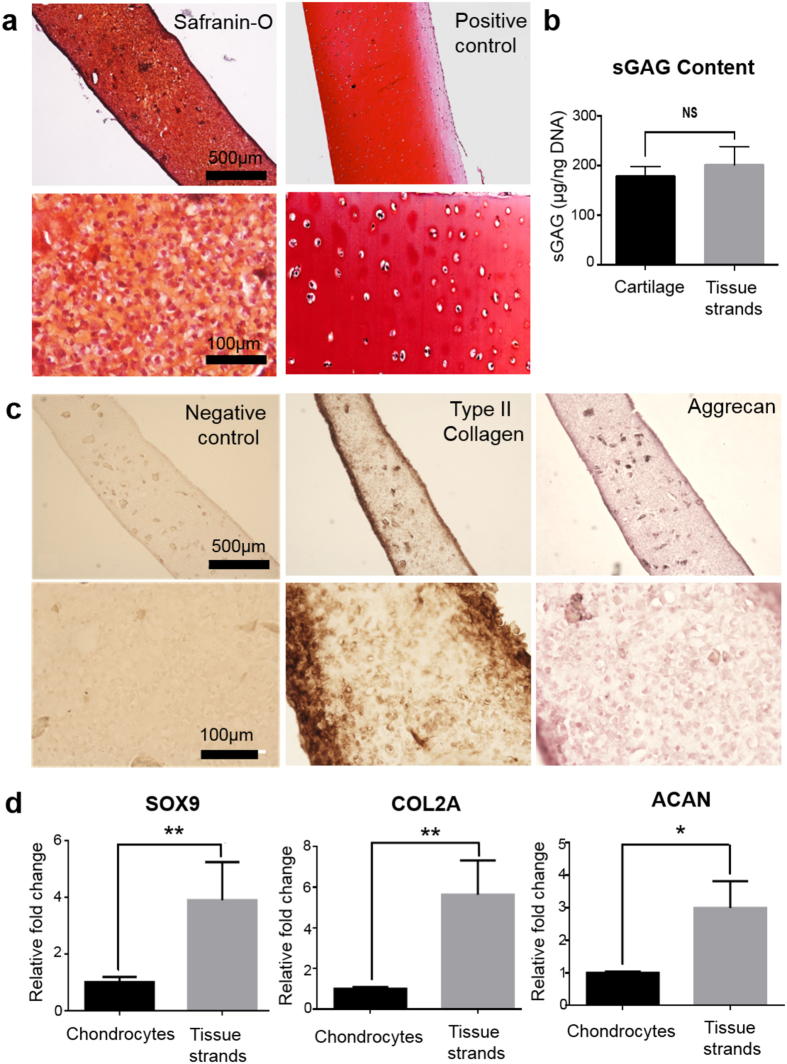
Functional and transcriptional characterization of tissue strands. (**a)** Functional evaluations of cartilage tissue strands showing positive staining of Safranin-O compared to native cartilage (positive control). (**b)** sGAG content measurement by DMMB assay type in comparison to native cartilage (control). (**c)** Positive type II collagen and Aggrecan staining compared to isotype IgG antibody (negative control). (**d)** Real-time PCR analysis of tissue strands demonstrating significant expression of cartilage-specific genes including Sox9, COL2A, and ACAN compared to monolayer-cultured bovine articular chondrocytes (*n*=3). All data presented as average ± SD unless otherwise stated.

Histological evaluation of cartilage tissue strands was carried out at the end of two weeks of *in-vitro* culture, and the images are presented in Fig. 4a. After two weeks of chondrogenic induction, a substantial amount of proteoglycan deposition was observed in the tissue strands with strong positive staining for safranin-O close to native cartilage (control). Safranin-O staining was homogeneously distributed throughout the entire tissue strand, and cells within tissue strands also displayed a characteristic cobblestone-like morphology. Cartilage tissue strands had higher cellularity, while native cartilage had relatively lower cell density and a higher ratio of ECM. Although cells within the tissue strands were not fully developed with their lacunae in two weeks, further maturation would grant them more differentiated characteristics like chondrocytes in native cartilage. In dimethylmethylene blue (DMMB) assay, sGAG content from tissue strands were 200.9 ± 21.69 μg/ng DNA, while native cartilage had sGAG content of 178.1 ± 11.45 μg/ng DNA (Fig. 4b). Tissue strands showed slightly higher proteoglycan production than native articular cartilage, while no significant difference was present between those two.

To further assess the properties of two-week cultured cartilage tissue strands, immunostaining was used to characterize the expression of cartilage-specific markers (type II collagen and Aggrecan) (Fig. 4c). Immunohistochemistry showed a significant amount of type II collagen (dark brown) positive staining as well as Aggrecan (dark purple) positive staining throughout tissue strands. Stronger staining for both markers was particularly observed at the edges due to early aggregation of cells near the luminal surface of the capsules. In isotype IgG antibody control, only the background color was observed, and no specific staining was visible at different magnitude. All these data demonstrated that chondrocytes in tissue strands could maintain their chondrogenic phenotype, forming cartilage tissue *in vitro*. Regenerated cartilage had characteristics that closely resembled the native cartilage tissue.

To further validate the potential of tissue strands for fabricating functional cartilage tissue, we examined the functionality of cultured cartilage tissue strands. Cartilage-specific genes and protein markers were assessed. Real-Time PCR gene expression analysis revealed relatively higher expression of cartilage-specific marker genes in tissue strands when compared to monolayer-cultured bovine articular chondrocytes (Fig. 4d). Sox9 a transcription factor required for maintaining the chondrocyte phenotype and during chondrogenesis showed a nearly four-fold change, which led to a significant increase in type II procollagen (COL2A) gene expression during chondrocyte differentiation. Effectively, the COL2A gene expression showed a nearly six-fold increase when compared to monolayer cultures, indicating that cells were actively making cartilage-specific protein to serve as ECM within tissue strands. Aggrecan (ACAN) gene expression was up regulated to nearly three-fold in tissue strands. These two molecules play an essential role in keeping the functional phenotype of articular chondrocytes, which further supports the hypothesis that tissue strand is an ideal environment for chondrogenesis.

### Bioprinting of Cartilage Tissue Strands

Upon characterization, we demonstrated the self-assembly characteristics of tissue strands, which grant them the capability to form larger tissues upon cellular fusion. To test the potential of tissue strands for self-assembling into a larger tissue patch, fusion experiments were carried out between matured cartilage tissue strands. In our study, fusion of cartilage tissue strands started as early as 12 hours during Day 1 (Supplementary Fig. 4) post-fabrication of strands. Further fusion was observed with more cell migration and ingrowth across each other on Day 4, when two strands were slightly contracted towards each other, with the edge lightly rounded up. On Day 7, strands were almost completely fused into a single tissue patch, with more contracted morphology and no visible gap between them. Fusion time depends on the maturity level of strands; more rapid fusion was observed when the strands were harvested from alginate capsules early.

**Figure 5:**
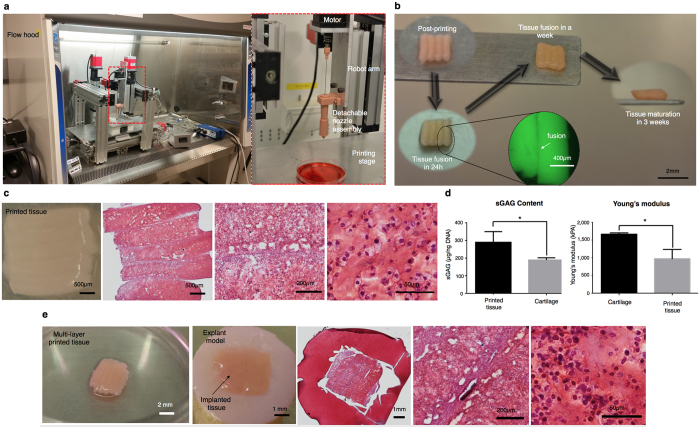
Bioprinting of cartilage tissue patches. (**a)** Left: The homemade MABP in a flow cabinet. Right: A new print head design loaded with detachable nozzle assembly for tissue strand printing. (**b)** Images of printed tissue morphology over 3 weeks of incubation. (**c**) Characterization of a bioprinted cartilage 3 mm × 3 mm tissue patch showing Safranin-O/Fast Green at different magnifications. (**d)** sGAG content (*n* = 3) and Young’s modulus (for compression) (*n* = 4) of the bioprinted cartilage. (**e)** Implantation of 3D printed tissue patches into a 4 mm × 4 mm osteochondral defect with 2 mm thickness showing Safranin-O/Fast Green histology examination at different magnifications. All data are presented as average ± SD unless otherwise stated.

To test the potential of using tissue strands as “bioink” for fabricating larger tissue patches, we performed experiments by directly bioprinting cartilage tissue strands into a defined shape without the need for a delivering medium during bioprinting (i.e., hydrogel or culture media) or a mold (i.e., agarose mold) post-bioprinting to ensure fusion and further aggregation. In order to bioprint tissue strands, we designed and customized a detachable nozzle system. Before bioprinting, tissue strands were loaded into the detachable nozzle under sterile conditions. The nozzle was then mounted onto a homemade Multi-Arm BioPrinter (MABP)^21^ (Fig. 5a), where highly long tissue strands were refilled whenever needed. Using the MABP and the customized print head (Supplementary Fig. 5 and Supplementary Methods S4), tissue strands were successfully bioprinted (Fig. 5b). Individually printed tissue strands attached to each other during the printing process because the strands were adhesive enough. In a day, the strands partially fused to each other as can be seen in the fluorescence image (Fig. 5b). They completely fused in a week and formed an integrated piece of maturated cartilage tissue three weeks post-bioprinting. Engineering tissue strands such as establishing their culture conditions is highly critical for bioprinting, while immature tissue strands could not be bioprinted successfully due to significant decomposition in their structure during extrusion (Supplementary Fig. 6). In Fig. 5c, a printed tissue (3 mm × 3 mm) is shown with Safranin-O staining revealing that the bioprinted tissue had a significant amount of proteoglycan formation and that the interface of each tissue strand was well integrated. Cells within fused tissue displayed a differentiated phenotype with rounded morphology, close to chondrocytes. Some delamination between strands were observed, which can be attributed to the shear stress exerted during sectioning process. The sGAG content of printed cartilage tissue was 289.7 ± 34.88 μg/ng DNA, while native cartilage had a sGAG content of 189.2 ± 7.355 μg/ng DNA (Fig. 5d, left). High sGAG content and cell density can be attributed to the large number of microinjected cells during strand fabrication as well as further proliferation of cells and maturation of the strands. The Young’s modulus (for compression) of printed cartilage was 1,094 ± 26.33 kPa, that of native cartilage was 1,664 ± 19.39 kPa (Fig. 5d, right). It can be expected that the mechanical properties of strands would get better if they were cultivated for a longer period of time under chondrogenic induction.

Multi-layers of tissue strands were also 3D bioprinted for implantation using a 90° lay-down pattern and exhibited tight integration both horizontally and vertically, as demonstrated in Fig. 5e. Tissue constructs were 3D bioprinted and able to fuse into one piece of cartilage tissue immediately after incubation as early as 12 hours, and maintained their integrity in culture. During two weeks of chondrogenic incubation, bioprinted tissues were completely fused and matured into an intact piece of cartilage with no visible gaps between layers. A cartilage defect repair study was then performed on a bovine osteochondral model. A 4 mm × 4 mm full thickness (2 mm) cartilage defect was made on each explant, and the printed cartilage was then implanted into the defect by press-fitting without using a bioglue. Explants were cultured under chondrogenic conditions for an additional four weeks. Tissue histology revealed that the implanted tissue exhibited proteoglycan-rich ECM. Although the implanted tissue adhered to the defect and stayed intact in it, histology results show limited integration with native tissue, as usually experienced in regenerative medicine therapies for articular cartilage lesions^22^. This can be attributed to the highly compact ECM of native cartilage^23^ as well as the bioprinted tissues and direct solid-to-solid interface of tissues (the explant and printed one) that constrains recruitment of migratory progenitor cells from the explant and migration of chondrocytes from the printed tissue. For future work, this can be however overcome by using a bioglue, such as an injectable composite fibrin-hyaluronic acid gel^24^, that can enable recruitment of native chondrocytes from the explant and facilitate better integration.

## Discussion

This study presents a first-time bioprinting of scaffold-free scalable tissue strands in a near-solid state, enabling fabrication of larger tissue patches that can be used for various purposes such as regenerative medicine and tissue engineering, tissue models for drug testing, and disease modeling. We demonstrated a novel approach in the microfabrication of scalable tissue strands as building units that do not need a liquid medium during printing or a molding enclosure for facilitating cell aggregation and fusion. Tissue strand diameter stabilizes and tissue strands preserve their cylindrical shape after 10-day culture (Fig. 2e), which enabled standardization of the presented 3D bioprinting system and ensured the repeatability of the bioprinting process. These tissue strands distinguish themselves from existing ones by not using any exogenous biomaterials, and by being derived entirely from the target tissue or organ. To our knowledge, bioprinting of larger-scale leak-free tissues in a short period of culture time using scaffold-free technology has not been demonstrated yet. Using the presented approach, bovine articular cartilage tissues were successfully fabricated with biochemical and mechanical properties close to native cartilage tissue (Figs 4 and 5). The implantation of bioprinted cartilage also underwent remolding with the host tissue in a bovine explant model; however, a bioglue can be applied to improve integration and further *in-vivo* implantation can be performed to evaluate its effectiveness and clinical potential. The success of this study may provide a novel platform for tissue or organ fabrication by taking advantage of the self-assembly ability of biological tissues during organogenesis.

Previous work by Forgacs and his coworkers^14^,^16^ demonstrated bioprinting of cell pellet (in strand form) into an agarose mold, which was also 3D printed in tandem with the cell pellet. The major hurdle with this approach is the difficulty of fabricating scale-up tissues without using a mold support as the mold structure restricts the cell pellet into a highly confined space^25^,^26^. Our results suggest that larger-scale tissues can be 3D bioprinted without any need for a mold during bioprinting process, which limits the size of bioprintable tissues restricting them to highly thin tubular shapes. As different tissues are comprised of different cells, further research is required to understand maturation and bioprintability of different tissue strands in order to apply the proposed approach for other tissue types.

Although a significant number of cells is required to prepare tissue stands, which is labor-intensive and costly as expanding these many cells is time consuming, the presented scaffold-free approach better facilitates cellular interactions and enables closer tissue biomimicry. On the other hand, in the traditional scaffold-based approach, less number of cells is required as cells are encapsulated in hydrogel matrix; however, subsequent ECM formation, degradation and digestion of the hydrogel matrix, and proliferation of cells are not trivial to control. In addition, cells are immobilized within hydrogels and do not spread and proliferate quickly. Immobilization of cells and limited cell-cell interactions slows down the maturation process. For example, Cui *et al*. demonstrated bioprinting of human chondrocytes in poly(ethylene glycol) dimethacrylate hydrogel^27^, where the maturation of free-standing tissue constructs during six-week culture period, evaluated by proteoglycan content, was significantly less than the level of maturation achieved in this work. For a detailed comparison of scaffold-based and scaffold-free bioprinting approaches, the reader is referred to our recent work^25^.

For future research, bioprinting vascularized tissues is one of the most critical steps in scaling up the currently bioprintable tissues to clinically relevant sizes, particularly for tissues and organs with a high-volumetric oxygen-consumption rate, such as cardiac, pancreas, or liver tissue. Bioprinting of these parenchymal tissues integrated with a vascular hierarchical network spanning arteries and veins down to capillaries^28^ will be a great bottom-up tissue fabrication strategy enabling future organ printing technologies for transplantation^1^. Therefore, bioprinting of pre-vascularized scalable mini-tissue units in tandem with bioprinting a macro-scale vascular network will be the next step towards scale-up tissue and organ printing.

## Methods

### Materials and alginate capsule fabrication

Prior to making a hydrogel solution, sodium alginate and calcium chloride (CaCl_2_) powder (Sigma Aldrich, United Kingdom) was treated with ultraviolet (UV) light for sterilization (three 30-minute cycles). Sterilized sodium alginate powder was dissolved in sterile deionized water to get a 4% (w/v) alginate solution. The alginate solution was subjected to magnetic stirring until reaching homogeneity. 4% CaCl_2_ solution was used as a crosslinking agent during the coaxial extrusion method (as detailed in Supplementary Fig. 1a), which facilitated the generation of tubular alginate capsules with uniform size and shape made of non-toxic, low-cost, and flexible alginate. The fabrication system consisted of a homemade coaxial nozzle unit (Supplementary Methods S1 for nozzle fabrication) connected to a pneumatic air dispenser (EFD Nordson, USA) and a mechanical pump (New Era Pump System Inc., USA) for alginate and CaCl_2_ extrusion, respectively. The coaxial nozzle assembly was composed of a 22 G inner nozzle (0.71 mm and 0.41 mm for outer and inner diameters, respectively) and a 14 G outer nozzle (2.11 mm and 1.69 mm for outer and inner diameters, respectively). The alginate-dispensing pressure was set at 82.7 kPa, while the CaCl_2_ dispensing rate was set at 16 ml/min. Pre-crosslinked alginate tubular capsules were extruded into a CaCl_2_ pool and left overnight for complete crosslinking.

### Cell preparation

The fresh stifle joints from young adult cattle (15–24 months old) were obtained from a local abattoir (Bud’s Custom Meats, Riverside, IA). Articular cartilage was harvested from the femur condyle and rinsed in Hank’s Balanced Salt Solution (HBSS, Life Technologies, California, USA) supplemented with 100 U/μl penicillin, 100 μg/ml streptomycin, and 2.5 μg/μl fungizone (Invitrogen Life Technologies, Carlsbad, CA). Full-thickness cartilage samples were minced into fine pieces and then digested overnight with 0.25 mg/ml collagenase type I and pronase E (1:1) (Sigma-Aldrich, St. Louis, MO) dissolved in culture medium in a shaking incubator overnight (0.25 mg/ml each). After isolation, primary chondrocytes were re-plated and cultured in Dulbecco’s modified Eagle’s medium (DMEM) and Ham’s F12 (1:1 mixture) supplemented with 10% fetal bovine serum (Life Technologies, Grand Island, NY), 50 μg/μl L-ascorbate, 100 U/μl penicillin, 100 μg/ml streptomycin, and 2.5 μg/μl fungizone at 37 °C with 5% CO_2_.

### Fabrication of Tissue Strands

Primary chondrocytes were expanded until the desired number was reached and were then harvested to make a large cellular mass. Cells were first suspended in culture medium, and centrifuged at 2,000 rpm for 10 minutes forming a cellular mass at the bottom of a 50 mL conical tube. The cellular mass (cell pellet) was then incubated at 37 °C with 5% CO_2_ overnight in DMEM-based media under static culture conditions with 2% fetal bovine serum, supplemented with 10 μg/μl penicillin, 10 μg/ml streptomycin, and 2.5 μg/μl Fungizone (Invitrogen Life Technologies, Carlsbad, CA), in order to have sufficient adhesive ability and mechanical integrity for further processing. The next day, cell pellet was aspirated by a customized syringe unit (Hamilton Company, Reno, NV) (Supplementary Fig. 2a) and was gently injected into tubular conduits. Tubular conduits were used as semi-permeable capsules for cell aggregation by tying ends with vascular clamps (Thomas Scientific, Swedesboro, NJ). Semi-permeable alginate capsules do not allow cells to move out and keeps them nutriented during the aggregation process. The injected pellet was incubated for seven days using the same cell culture medium, which was changed every two days. Next, alginate was dissolved in a 1% sodium citrate solution in dH_2_O (Sigma Aldrich, USA) for five minutes, leaving pure cellular tissue strands with acceptable cohesiveness to handle for transferring. As expansion of chondrocytes to large quantities may induce lost of chondrogenic phenotype of the harvested cells, we further cultured tissue strands under chondrogenic conditions to maintain their phenotype.

### Cell viability assay

Cell viability assay was carried out by live/dead staining per the manufacturer’s instructions. Cell calcein acetoxymethylester (calcein AM) and ethidiumhomodimer-2 (Invitrogen Life Technologies, Carlsbad, CA), at a concentration of 1.0 mM each, was used. Calcein AM labels living cells in bright green fluorescent. Ethidium homodimer is a red fluorophore that stains non-viable cells but cannot penetrate living cells. Each sample was washed with HBSS before staining. After 30 minutes of incubation, samples were imaged using an Olympus FluoViewTM FV1000 laser scanning confocal microscope (LSCM) (Olympus NDT Inc., MA). Z-axis projections were assembled from images of each sample from surface to bottom with a depth of 1,000 μm at 20-μm intervals. ImageJ software (National Institutes of Health, Bethesda, MD) was used for automated quantification of the intensity of red- and green-stained tissue strands.

### Uniaxial tensile testing for tissue strands

Cartilage tissue strands cultured under chondrogenic condition at different time points (1 w, 2 w, 3 w) upon releasing from capsules were used for mechanical property evaluation. A tensile testing machine (MTS Systems Corporation, Eden Prairie, MN) was used to perform uniaxial tensile testing on all samples (Supplementary Fig. 6a). Briefly, tissue strands were fixed on customized grips at both ends and rigidly held by a 5N load cell and a platform on the machine. Tissue strands were manually loaded until reaching positive tension, and sample diameter and original length were measured with a digital caliper at this point. After that, tensile loads were applied to tissue strands at a 0.1 mm/s loading rate until they broke. Tensile stress and strain were recoded as well as ultimate tensile strain. Young’s modulus was calculated based the slope of the stress/strain curve. Each group was tested for 3–4 samples, and optimal mechanical properties were determined based on culture time. Tissue strands with appropriate mechanical properties were used for the bioprinting study.

### Histology and immunohistochemistry analysis

Cultured samples were fixed in 4% paraformaldehyde, frozen and sectioned prior to histological evaluation. Sections underwent haematoxylin and Safranin O-fast green staining according to standard protocols^29^. For immunohistochemical analysis, 10 μm sections were incubated for 30 minutes in blocking solution to prevent nonspecific binding and were then incubated with primary antibodies overnight at room temperature. Rabbit anti-human polyclonal antibodies against collagen type II and Aggrecan (Developmental Studies Hybridoma Bank, Iowa City, IA) were used in this study. A goat anti-mouse secondary antibody (Vector Laboratories Inc., Burlingame, CA) was used for detection. The reaction products were visualized by the Vectastain ABC kit and the DAM Peroxidase Substrate Kit (Vector laboratories Inc., Burlingame, CA), according to the manufacturers’ instructions. All negative controls were done using the same staining without using primary antibodies. Observation was performed with an Olympus BX60 microscope (Olympus NDT Inc. Center Valley, PA).

### DMMB assay for sGAG content evaluation

sGAG content was determined by 1,9-dimethylmethylene blue (DMMB) dye-binding assay. Briefly, serially diluted samples were prepared and the DMMB solution was added. The absorbance was measured at 530 nm using the VMax Kinetic ELISA microplate reader (Molecular Devices, Inc., Sunnyvale, CA). sGAG content was normalized to DNA content in each specimen and presented as sGAG per cell. DNA quantification was also carried out. Briefly, two-week-cultured tissue strands as well as native articular cartilage were digested in the papain buffer and then subjected to DNA quantitation assay. A Quant-iT^TM^ PicoGreen dsDNA Assay Kit (Molecular Probes Inc., Eugene, OR) was used according to the manufacturer’s instructions. Fluorescence intensity was determined by a SpectraMax multidetection microplate reader (Molecular Devices, Inc., Sunnyvale, CA), using the wavelength of 480 nm (excitation) and 520 nm (emission). sGAG content from each sample was normalized to dsDNA content.

### Total RNA extraction, reverse transcription, and real time-PCR analysis

To check cartilage-tissue-specific gene-expression levels, tissue strands were homogenized in TRIzol reagent (Life Technologies, Carlsbad, CA), and total RNA was extracted using the RNeasy Mini Kit (QIAGEN, Valencia, CA) according to the manufacturer’s instructions. cDNA was reverse transcribed using TaqMan Micro RNA reverse transcription kits (Life Technologies, Carlsbad, CA) according to instructions from the vendor. SYBR Green Real-Time PCR kit (Life Technologies, Carlsbad, CA) was used to analyze the transcription levels of cartilage-matrix-related genes, including collagen type II, Aggrecan, and chondrogenic transcription factor Sox9. The reader is referred to Supplementary Methods S5 for details of real-time PCR.

### Self-assembly of tissue strands

Fusion experiments were carried out to test the potential of tissue strands to self-assemble into larger tissues. Briefly, multiple individual constructs were placed onto a 150 mm petri dish close to each other with contact. A minimum amount of culture media was supplemented, ensuring cells survive without losing their contact. Microscopic images (Leica Microsystems Inc., Buffalo Grove, IL) were taken at different time points to monitor the fusion process with minimal disturbance. For fluorescence microscopic images, cells were stained with Calcein AM (Life Technologies, Carlsbad, CA) prior to imaging.

### 3D bioprinting of tissue strands

To test the potential of using tissue strands as “bioink” for fabricating larger tissue patches, we performed experiments by directly bioprinting cartilage tissue strands into defined shapes, which later matured *in vitro* into integrated cartilage tissue. In order to bioprint tissue strands, we designed and customized a nozzle system and fabricated it by 3D printing as outlined in Supplementary Methods S4. Before bioprinting, tissue strands were loaded into the foldable nozzle under sterile conditions as demonstrated in Supplementary Video 1. Tissue strands were transferred into the nozzle within their capsules and placed into the nozzle cavity. Then, 4% sodium citrate solution was added onto the capsule to decrosslink it followed by removing the vessel clips. Next, the excess amount of sodium citrate solution was aspirated leaving some to keep the tissue strand hydrated. For longer strands, droplets of cell medium were added to prevent dehydration as bioprinting of longer strands took longer time. Finally, the foldable nozzle was mounted onto the MABP. We succeeded bioprinting tissue strands on both glass slides (see Supplementary Video 2) and filter papers in a dish. Prior to bioprinting on a filter paper, we hydrated the filter paper with droplets of media to keep the surface and the bioprinted tissue strands hydrated. A robot printing speed of 100 mm/min and an extrusion speed of 50.8 mm/min were used. No support material was used during or after bioprinting, and tissue strands sufficiently adhered on the surface of the filter paper preventing slippage during bioprinting. Further droplets of media were added to keep them hydrated for a few hours until sufficient tissue integrity was achieved. Later, samples were transferred to a tissue culture dish for incubation.

### Biomechanical assessment for the quality of regenerated cartilage tissue

To further characterize the mechanical property of regenerated cartilage tissue, we performed compression tests on printed tissue patches using an MTS Insight materials testing machine (MTS Systems Corporation, Eden Prairie, MN) (Supplementary Fig. 7b). Briefly, printed tissue thickness was measured by a laser measurement system (Keyence Corporation of America, Itasca, IL). A non-porous platen was brought into contact with the tissue surface, and the tissue was compressed to 20% strain at 2 mm/s (human walking) with a 10 N load held for 5 minutes. The Young’s modulus was calculated during the initial loading period (between 15% and 20% strain). Native cartilage samples were harvested from the trochlear groove of bovine femur condyles and were used as controls.

### Implantation of bioprinted cartilage patches

To test the potential of bioprinted cartilage for repairing articular cartilage injuries, a tissue implantation study was performed on a bovine *in-vitro* cartilage defect model. Briefly, osteochondral explants (12 mm in diameter and 8–10 mm in thickness) were harvested from the groove of bovine femur condyle heads (12–18 months of age, 8 animals in total) by a customized drill bit. All explants were cultured in DMEM supplemented with 10% fetal bovine serum (Invitrogen Life Technologies, Carlsbad, CA), 50 μg/ml L-ascorbic acid, 100 U/ml penicillin, 100 μg/ml streptomycin, and 2.5 μg/ml fungizone. After two days of pre-equilibrium culture, full-thickness (2 mm) square chondral defects (4 mm × 4 mm) were created as previously described^30^. The printed cartilage tissue was then implanted into the defect by press-fitting. After tissue implantation, explants were placed back in culture in chondrogenic medium (DMEM containing 10 ng/ml TGF-β1, 100 ng/ml IGF-1, 0.1 μM dexamethasone, 25 μg/ml L-ascorbate, 100 μg/ml pyruvate, 50 mg/ml ITS+ Premix) at 5% CO_2_, 37 °C for up to four weeks. Histology and immunohistochemistry were used to evaluate cartilage-specific markers expression and ECM formation.

### Statistical Analysis

All data are presented as the mean ± SD and were analyzed by GraphPad Prism 6 (GraphPad Software, La Jolla, CA) using Student’s t-test. Differences were considered significant at p < 0.05 (*) and p < 0.01 (**). NS stands for “not significant.” The percentage of viable cells for each experimental group was calculated by averaging the values of three different locations from three different samples.

## Additional Information

**How to cite this article**: Yu, Y. *et al*. Three-dimensional bioprinting using self-assembling scalable scaffold-free “tissue strands” as a new bioink. *Sci. Rep*. **6**, 28714; doi: 10.1038/srep28714 (2016).

## Supplementary Material

Supplementary Information

Supplementary Video S1

Supplementary Video S2

## Figures and Tables

**Figure 1:**
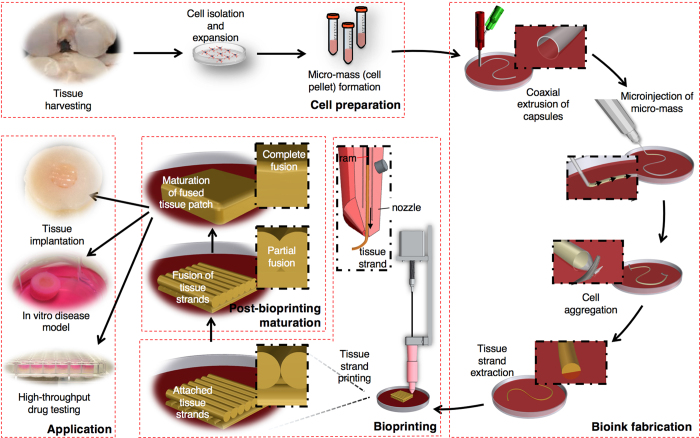
Schematic elucidating the concept of tissue printing using tissue strands as a new bioink. Cells extracted from harvested tissues in large numbers are microinjected into tubular capsules, where tissue strands are obtained through aggregation of cells. Upon fabrication, scaffold-free tissue strands are printed without need for a delivering medium or a support structure for cell aggregation and tissue fusion. Printed tissue constructs are cultivated for fusion and further maturation, which can be used for various applications such as tissue engineering and regenerative medicine, *in-vitro* disease models, or drug screening.
