# Supplementary material for: Three-dimensional bioprinting using self-assembling scalable scaffold-free “tissue strands” as a new bioink
Source: Sci Rep. 2016 Jun 27;6:28714. doi: 10.1038/srep28714 (PMC4921918; doi:10.1038/srep28714)
Supplement: Supplementary Information [file srep28714-s1.pdf]

Supplementary Information for: Three-dimensional bioprinting using self-assembling scalable "tissue strands" as a new bioink

Yin Yu[1, 2], Kazim K. Moncal[3, 4], Jianqiang Li[5], Weijie Peng[3], Iris Rivero[5], James A. Martin[6], Ibrahim T. Ozbolat[3, 4, *]

[1]Harvard Medical School, Harvard University, Cambridge, MA, USA

[2]The Center for Engineering in Medicine, Massachusetts General Hospital, Boston, MA, USA

[3]Engineering Science and Mechanics Department, The Pennsylvania State University, State College, PA, USA

[4]Huck Institute of Life Sciences, The Pennsylvania State University, State College, PA, USA

[5]Industrial and Manufacturing Systems Engineering, Iowa State University, Ames, IA, USA

[6]Department of Orthopaedics and Rehabilitation, The University of Iowa, Iowa City, IA, USA

[*]Email: ito1@psu.edu

Supplementary Methods

S1. Manufacturing of the coaxial nozzle unit

This nozzle assembly consists of a feed tube, an outer tube, and an inner tube, as shown in Supplementary Fig. 1a. The feed tube was used to feed alginate into the cavity formed between the outer and inner tubes (sheath section) while the crosslinker (calcium chloride) was fed through the inner tube (core section) to create tubular capsules. A hole with the same outer diameter as the feed tube was created in the barrel of the outer tube to attach the feed tube. The luer lock hub on the barrel of the outer tube was removed using a lathe, and the tip was ground to ensure the inner and the outer dispensing tips were even. The feed tube was inserted until it was flush with the inner diameter of the barrel. The two tubes were aligned concentrically using a stainless steel fixture manufactured using micro-milling. Then, the tubes were assembled using laser welding.

S2. Dimensional analysis of tissue strands in alginate capsules

Upon microinjection of cells into alginate capsules, microscopic images of tissue strands were taken daily to observe formation of tissue strands. The morphology of tissue strands was monitored using an inverted microscope (Leica Microsystems Inc., Buffalo Grove, IL) equipped with a digital camera, and dimension measurements were conducted using NIH Image J software (National Institutes of Health, Bethesda, MD).

S3. Scanning electron microscopy imaging

Field emission scanning electron microscopy (SEM) (Hitachi S-4800, Japan) was used to investigate the ultra-morphology of cartilage tissue strands. After dissolving alginate capsules, tissue strands were kept for 1 to 4 weeks culture time and then fixed in 4% paraformaldehyde (Sigma Aldrich, USA) for one hour and then dehydrated in graded ethanol solutions (50 to 100%). Afterwards, samples were chemically dried with hexamethyldisilazane (Sigma Aldrich, USA), sputter-coated (K550 Emitech Sputter Coater) (Quorum Technologies Limited, UK), and then observed at an accelerating voltage of 1.5 kV.

S4. High resolution 3D printing of prototype nozzle units for tissue strand printing

In order to bioprint tissue strands, we designed and customized nozzle system, and fabricated it using a high-resolution top-down stereolithography 3D printing system (Perfactory® Micro HiRes 3D Printer (envision TEC, Germany)). The print head was composed of four main parts: two plastic folding shells, a metal barrel and a step-motor system. Briefly, 3D computer-aided design

(CAD) files for the nozzle design was generated by PTC Creo Prarmetric, as demonstrated in Supplementary Fig. 5b, and was 3D printed using E-Shell 200 series biocompatible polymer material (envision TEC, Germany) allowing tight interlock. A high-precision stainless steel barrel (800µm) was customized to the length fitting the nozzle with an internal diameter of 808 ± 8 µm (considering the error less than %1 according to manufacturer's datasheet). A connector was also 3D printed using the same materials for assembling the metal barrel to the step motor system. Before bioprinting, tissue strands were loaded into the foldable nozzle under sterilize condition followed by mounting the print head onto the MABP. In addition, a detachable porous cartridge was designed and 3D printed that enabled rapid and practical loading and unloading of tissue strands within cartridges, where an interlocking system provided ease of fixing the cartridge when the cartridge is rotated inside the nozzle (Supplementary Fig. 5c). Porous cartridges were designed to enable culturing alginate capsules in them (facilitating media flow in and out), decrosslinking of alginate capsules (leaving tissue strands in them), and loading of cartridges into the nozzle head easily.

S5. Real-time PCR analysis

Cartilage-specific gene expression (collagen type II, aggrecan, and Sox9)[1] was measured by real-time PCR in monolayer-cultured bovine articular chondrocytes and cartilage tissue strands. Collagen type II is the basis for articular cartilage, and the collagen type II gene is a marker related to chondrocyte phenotype and function. Aggrecan, also known as cartilage-specific proteoglycan core protein (CSPCP), is a protein that is encoded by the ACAN gene in humans. The encoded protein is an integral part of the extracellular matrix in cartilage tissue. Sox-9 is a transcription factor related to chondrogenic differentiation, which is the main function of CPCs. PRG-4 is a gene encoded for proteoglycan 4 or lubricin, which is a protein that acts as an articular joint cell lubricant. The relative fold change in the qPCR study was the expression ratio derived from $2^{-\Delta\Delta Ct}$, where $-\Delta\Delta Ct = \Delta Ct_{reference} - \Delta Ct_{target}$. Supplementary Table 1 provides information about the primers used in real-time PCR analysis.

Supplementary Tables

Supplementary Table T1: Primer information for real-time PCR for B-actin, collagen type-II, aggrecan, and Sox-9.

Gene		Primer
β-actin	Forward:	5'- TCGACACCGCAACCAGTTCGC -3'
	Reverse:	5'- CATGCCGGAGCCGTTGTCGA -3'
Aggrecan	Forward:	5'- ACCAGACAGTCAGATACC -3'
	Reverse:	5'- GCAGTAGACATCGTAGG -3'
COL2A	Forward:	5'- AAGACGCAGAGCGCTGCTGG -3'
	Reverse:	5'- GCAGTAGACATCGTAGG -3'
Sox9	Forward:	5'- CGGTGGTGTTTGGCCATGTAATGA -3'
	Reverse:	5'- GAGAGAGGGGAGTCCTATCCTGGT -3'

Supplementary Figures

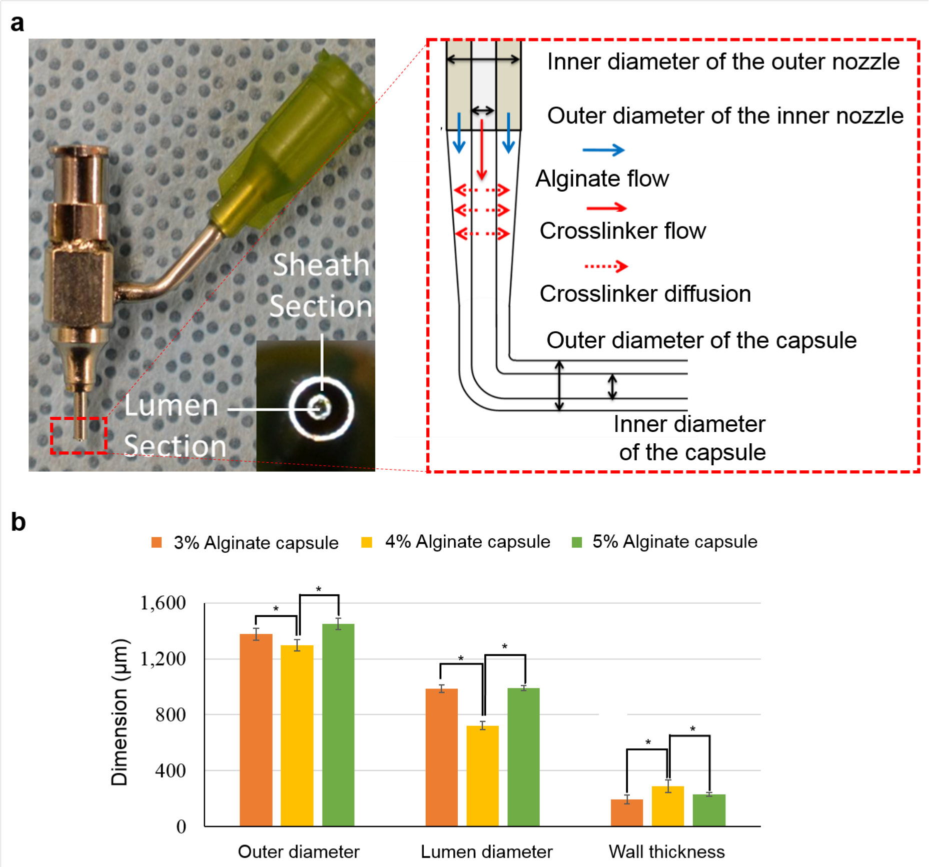

Supplementary Figure 1: Coaxial extrusion of alginate capsules. (a) The coaxial nozzle apparatus for extrusion of alginate capsules, where the crosslinker solution flows through the core section of the nozzle, contacting and diffusing through alginate, which is ejected through the sheath section of the nozzle. Coaxial-extrusion process generates highly long tubular capsules (more than a few meter-long) and (b) dimensions can be controlled by controlling alginate concentration and other parameters (i.e., crosslinker flow rate and nozzle configuration as presented in our earlier work[2]) (* denotes statistical significance at $p<0.05$).

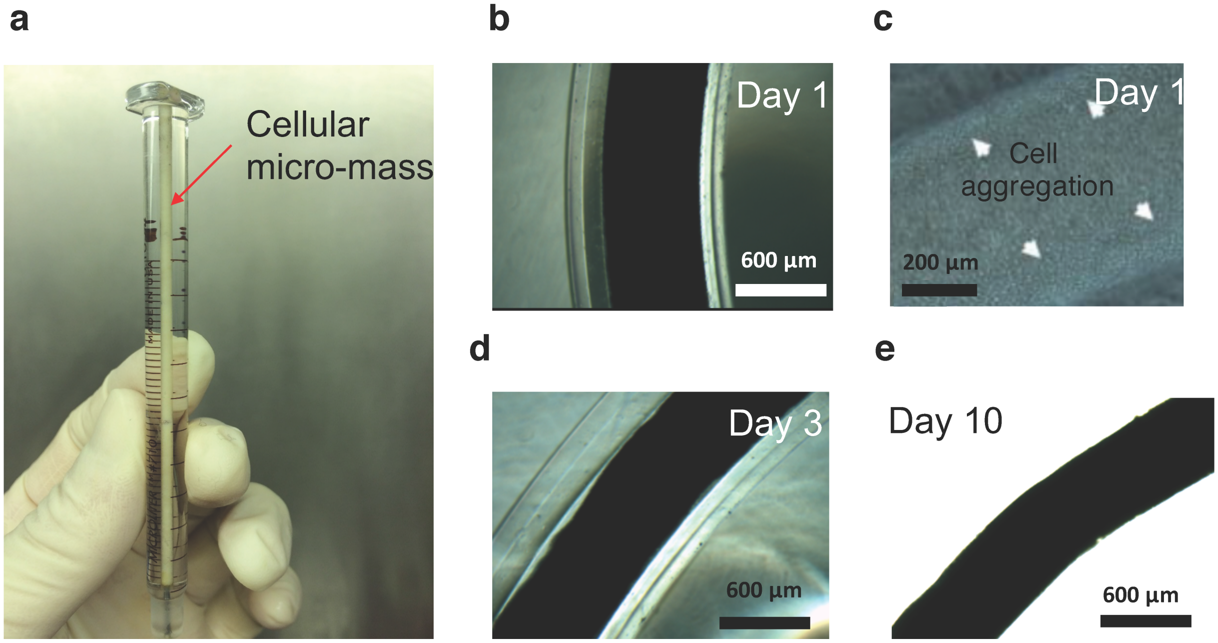

Supplementary Figure 2: Injection of cellular micromass and changes in the diameter of cartilage tissue strands during culture: (a) After microinjection process using a gas-tight syringe, (b) cell pellet filled the capsules completely without any visible space between the pellet and the capsule inner wall and (c) aggregation started at the outer section of the strands in 12 hours. (d) In three days, strands contracted significantly to 639 ± 47 µm in diameter, which further diminished to 507 ± 18 µm on Day 10 (e). The contraction of maturating tissue strands is mainly due to the normal contraction of healthy cultures of stromal cells in a 3D environment and the contractile potential of cartilage and the high cell-to-cell interaction in 3D culture system. We also evaluated contraction of mouse insulinoma beta cells in capsules and observed very minimal contraction (data not shown here) while parenchyma cells deposit limited ECM[3].

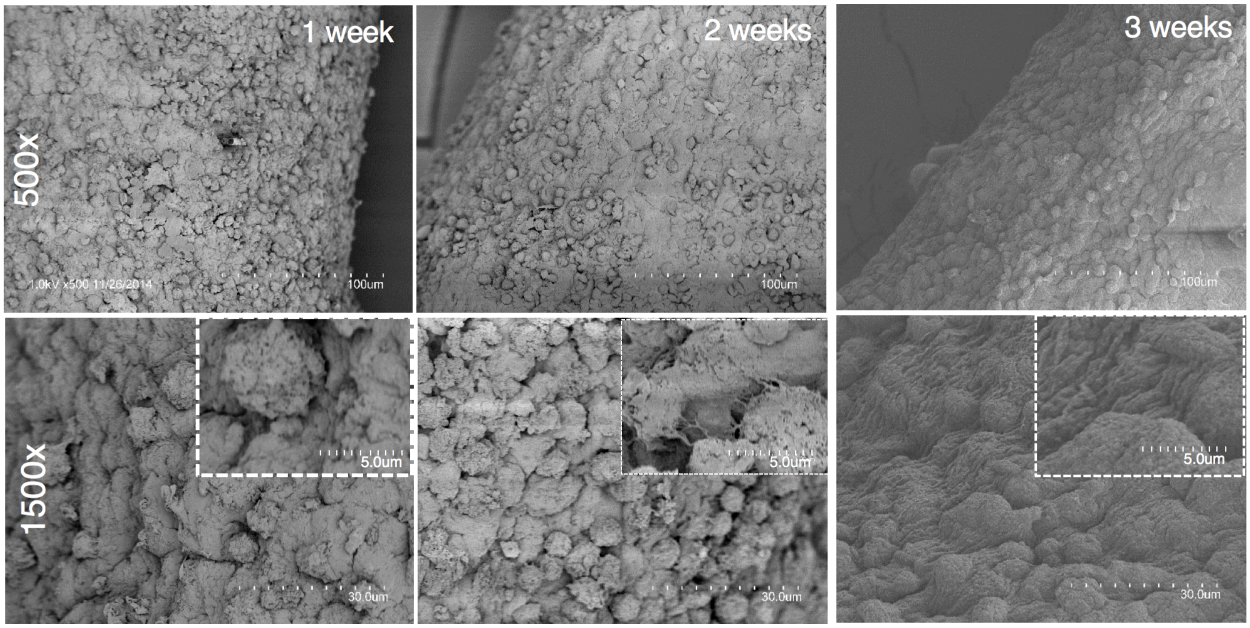

Supplementary Figure 3: SEM micrographs of cartilage tissue strands. Showing ultra-structure and morphology periodically over three-week culture time, where chondrocytes proliferated and deposited considerable amount of ECM. Tissue strands deposited fibrous ECM components, which were visible after two-week culture and increased over time. Increased fibrous ECM components such as collagen type II enhanced the mechanical properties of the strands as validated with the tensile stress test results presented in Fig. 3.

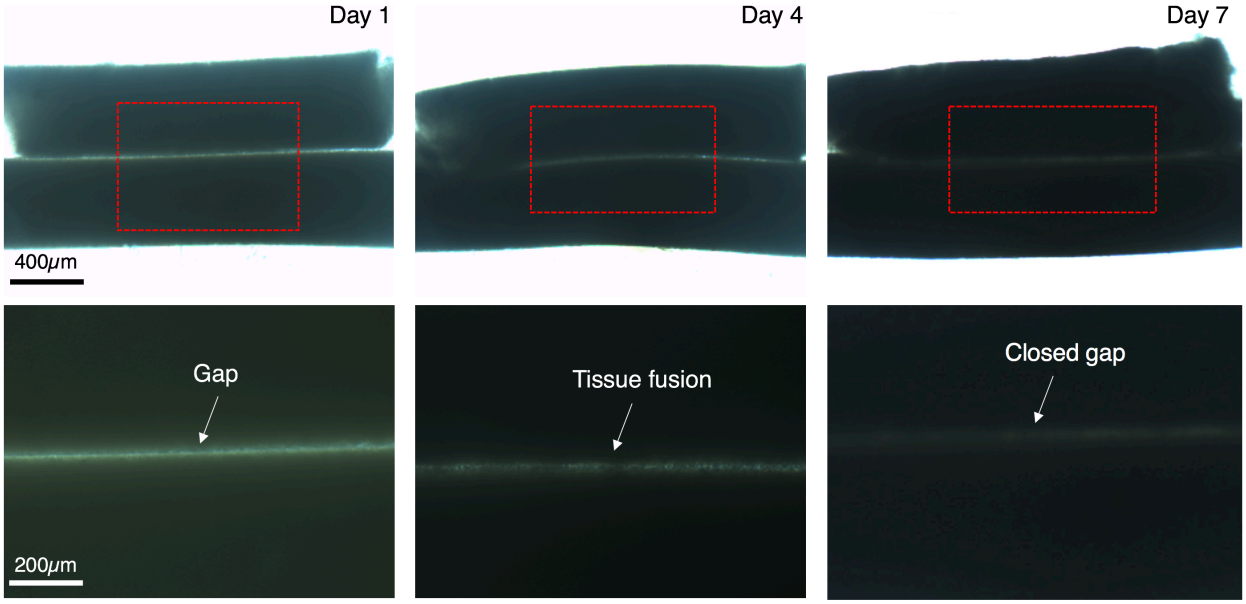

Supplementary Figure 4: Demonstration of self-assembly. When placed next to each other, fusion of tissue strands started in 12 hours on the first day and completed fully in 7 days in culture with the physical gap closed under microscope.

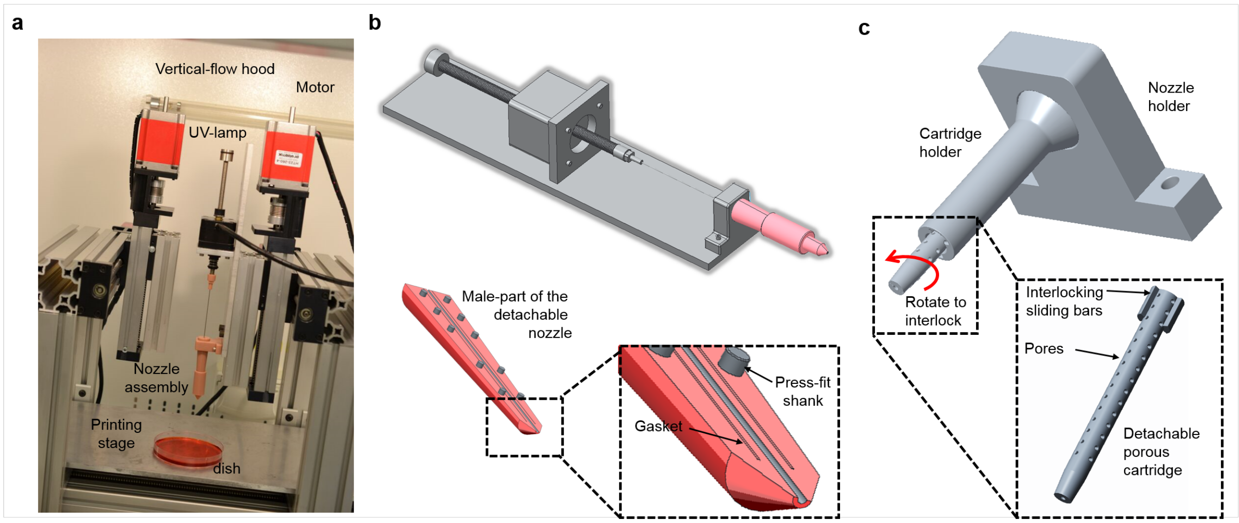

Supplementary Figure 5: Bioprinting mechanism for tissue strand bioprinting. (a) Setup for the in-house bioprinter, where the bioprinter is placed in a vertical flow hood (Air Science, Fort Myers, FL) with a prototype custom-made nozzle head. (b) Bioprinting head with a detachable nozzle assembly, where tissue strands can be place within nozzle cavity surrounded by gaskets for tight assembly and leak prevention when press-fit shanks on the male part engages into the hole in the female part. (c) A detachable porous cartridge design enabling rapid and practical loading and unloading of the cartridge using interlocking system on the cartridge.

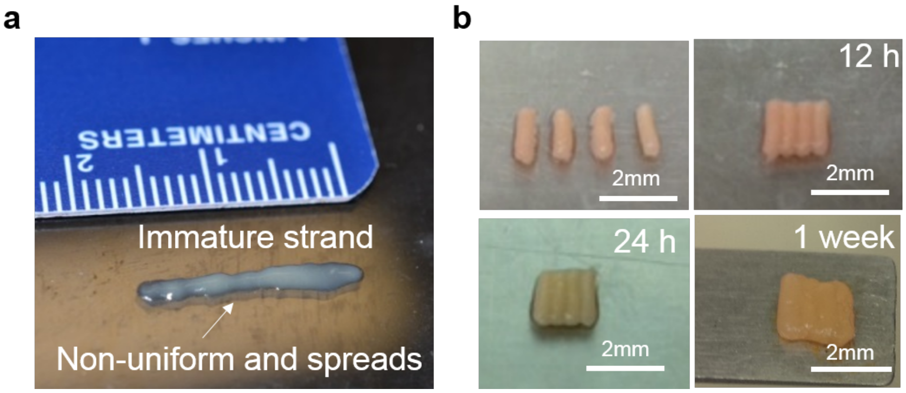

Supplementary Figure 6: Bioprinting of tissue strands. (a) Bioprinting of a cartilage strand in immature form, which was released from the capsules before full aggregation completed (particularly in the core section of the strand as the cells in the core aggregated later). During bioprinting, strands in semi-liquid form were structurally deformed and deposited, however, the deposited strand was not keep its integrity while it spread quickly due to its wettability. Once the bioprinted strand was exposed to the culture media, it dissolved in the media and disappeared, which necessitates confining and molding cell aggregates after bioprinting. (b) Tissue strands with enough maturity could be printed with preserving its shape and intactness resulting in successful deposition of strands next to each other. Upon contact, tissue strands attached each other due to their cohesive surface and fused in 12 hours followed by further maturation into a single cartilage tissue patch in a week. This demonstrates that the culture time for tissue strands is highly critical in order to successfully bioprint them.

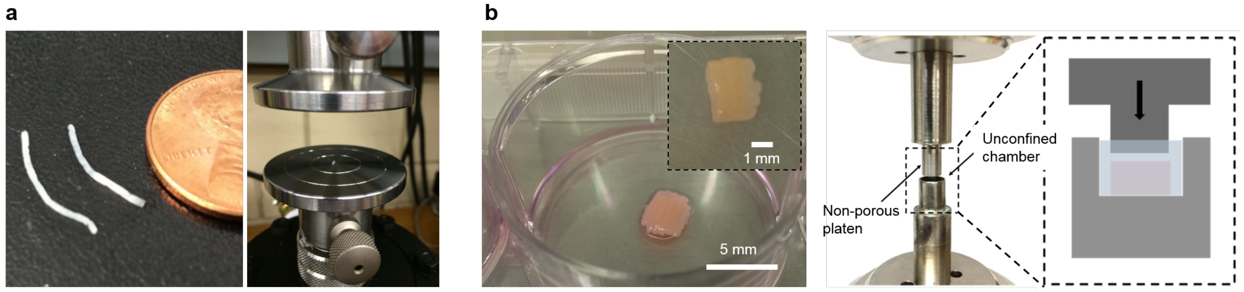

Supplementary Figure 7: Mechanical testing experiments. (a) Left: Maturated cartilage tissue strands used in tensile testing experiments. Right: A tensile testing machine (MTS Systems Corporation) was used to perform uniaxial tensile testing to obtain data for ultimate strength, Young's modulus and failure strain for one week, two weeks and three weeks cultured strands. (b) Left: 3D Bioprinted cartilage tissue samples three weeks post-bioprinting were trimmed (dashed inset) to fit into device to test Young's modulus (for compression). Right: Apparatus, an MTS Insight materials testing machine (MTS Systems Corporation), and the scheme (dashed inset) for stress-relaxation test.

Supplementary Videos

Supplementary Video 1: Loading of a tissue strand. A tissue strand was loaded into a foldable nozzle within its capsule followed by decrosslinking of the capsule. This way, the tissue strands was successfully loaded into the nozzle cavity. Note that tissue strand in this video was fabricated using rat heart microvascular endothelial cells (VEC Technologies Inc).

Supplementary Video 2: Extrusion and bioprinting of a tissue strand. A loaded tissue strand was extruded using a mechanical extrusion mechanism. Bioprinting of a tissue strand in solid form was successfully performed on a coverslip. No delivery medium was used and the tissue strand was printed in intact shape without any sign of spreading.

References

1. Bell, D.M. et al. SOX9 directly regulates the type-ll collagen gene. *Nat. Genet.* **16**, 174-178 (1997).
2. Zhang, Y., Yu, Y., Chen, H. & Ozbolat, I.T. Characterization of Printable Cellular Micro-fluidic Channels for Tissue Engineering. *Biofabrication* **5**, 024004 (2013).
3. Bataller, R. & Brenner, D.A. Liver fibrosis. *J. Clin. Invest.* **115**, 209-218 (2005).
